# ‘To me, it's ones and zeros, but in reality that one is death’: A qualitative study exploring researchers' experience of involving and engaging seldom‐heard communities in big data research

**DOI:** 10.1111/hex.13713

**Published:** 2023-01-24

**Authors:** Piotr Teodorowski, Sarah E. Rodgers, Kate Fleming, Naheed Tahir, Saiqa Ahmed, Lucy Frith

**Affiliations:** ^1^ Department of Public Health, Policy & Systems University of Liverpool Liverpool UK; ^2^ National Disease Registration Service NHS Digital Liverpool UK; ^3^ ARC NWC Public Advisor Liverpool UK; ^4^ Department of Law University of Manchester Manchester UK

**Keywords:** big data, PPI, public engagement, public involvement, qualitative, seldom‐heard

## Abstract

**Background:**

Big data research requires public support. It has been argued that this can be achieved by public involvement and engagement to ensure that public views are at the centre of research projects. Researchers should aim to include diverse communities, including seldom‐heard voices, to ensure that a range of voices are heard and that research is meaningful to them.

**Objective:**

We explored how researchers involve and engage seldom‐heard communities around big data research.

**Methods:**

This is a qualitative study. Researchers who had experience of involving or engaging seldom‐heard communities in big data research were recruited. They were based in England (*n* = 5), Scotland (*n* = 4), Belgium (*n* = 2) and Canada (*n* = 1). Twelve semistructured interviews were conducted on Zoom. All interviews were audio‐recorded and transcribed, and we used reflexive thematic analysis to analyse participants' experiences.

**Results:**

The analysis highlighted the complexity of involving and engaging seldom‐heard communities around big data research. Four themes were developed to represent participants' experiences: (1) abstraction and complexity of big data, (2) one size does not fit all, (3) working in partnership and (4) empowering the public contribution.

**Conclusion:**

The study offers researchers a better understanding of how to involve and engage seldom‐heard communities in a meaningful way around big data research. There is no one right approach, with involvement and engagement activities required to be project‐specific and dependent on the public contributors, researchers' needs, resources and time available.

**Patient and Public Involvement:**

Two public contributors are authors of the paper and they were involved in the study design, analysis and writing.

## 1 INTRODUCTION

Patient and public involvement and engagement (PPIE) has become embedded in health research and within the NHS,^1^ and is used in healthcare services^2^ to put the public perspective at the centre of the discussion^3^ and improve professionalism among medical practitioners.^4^ It helps to align priorities shared by researchers and the public^5^ and it helps researchers understand the lived experience of patients and the public.^6^ There is also an ethical argument that those who pay (taxpayers) should have a say on how their services and research are shaped.^7^ We follow the National Institute for Health and Care Research (NIHR) definition of public involvement and engagement.^8^ Public involvement in research means that work is ‘being carried out “with” or “by” members of the public rather than “to,” “about” or “for” them’. We use the term ‘public contributor’ to describe this role. Conversely, public engagement stands for activities ‘where information and knowledge about research is provided and disseminated’.

### 1.1 Big data

There are multiple definitions of big data in the literature.^9^ In this paper, we define big data research as reusing routinely collected medical data for research purposes. This can happen by linking large medical data sets from various sources. When initially collecting medical data, the public (or the researcher) might not be aware that their data may be later reused for research. Many big data research studies use opt‐out consent, where patients need to inform someone, usually their medical provider, that they do not want their medical data to be reused for research.

Public support is needed for these projects to be able to take place,^10^ and a systematic review has shown that the public generally supports the reuse of their medical data.^11^ However, they can be concerned that their data might be misused, for example, sold to private companies.^12^ PPIE can assist in alleviating these concerns.^13^ Hill et al.^14^ found that talking about and explaining the research process around big data improved their study participants' support in reusing their medical data. Public contributors can also contribute to the decision process on who can access medical data for research purposes, thus ensuring that a social licence exists.^15^ Social licence is more than meeting legal requirements and requires public trust that researchers will conduct their work ethically.^13^ Poor governance can lead to a deterioration of the social licence.^16^

### 1.2 Seldom‐heard communities

In addition to the ‘usual’ public, it is important to capture the voices of groups in our communities who are less frequently heard. Successful PPIE requires the inclusion of seldom‐heard communities,^5^, ^17^, ^18^ and researchers should aim to include them,^19^ but how to do it in a meaningful way remains challenging.^20^, ^21^, ^22^ Such communities are often easy to ignore, but not including them can make research findings ungeneralizable to all parts of society and miss the nuances of experiences specific to those groups^23^ and will not provide solutions for all communities.^24^ PPIE should be inclusive of and accessible to everyone.^5^ Not including seldom‐heard voices can reflect the power structures at play and perpetuate health inequalities. This is important as these communities might experience poorer social and health outcomes. For example, the Covid‐19 pandemic disproportionately affected people from ethnic minorities.^25^

The terminology and definitions in this area are contested. Some of the terms used include hard‐to‐reach,^23^, ^26^ seldom‐heard,^27^, ^28^ seldom‐listened,^29^ peripheral voices,^30^ marginalized^31^, ^32^ and underserved.^33^ The key characteristic of these definitions is that these communities are less included in research than other groups in mainstream society. Within the UK legal context, the Equality Act 2010 uses the term ‘protected characteristics’. These are age, disability, gender reassignment, marriage or civil partnership, pregnancy and maternity, race, religion or belief, sex and sexual orientation. The Act provides antidiscrimination laws and embeds requirements for diversity and inclusion for public bodies but is not always directly applied to research. However, it can be influential in how researchers approach diversity in their work.^18^ We will use ‘seldom‐heard’ as this shifts the responsibility for inclusion to researchers rather than blaming the public, as implied by the ‘hard‐to‐reach’ wording. Their inclusion (or a lack of it) is not a fault of these communities.^33^ When presenting the results, we kept the original terms used by participants when quoting them. However, we recognize that use of any terms might not necessarily represent how these communities would like to be described.

### 1.3 Research aim

Despite understanding the importance of PPIE, there is limited knowledge of how this can be effectively facilitated in big data research.^34^ A previously published system logic model identifying key elements of PPIE in big data research recognized the inclusion of seldom‐heard communities as a key component,^34^ and therefore, there is a need to understand how to ensure all voices are included.

This paper explores researchers' experiences of involving and engaging seldom‐heard communities in big data research.

## 2 METHODS

### 2.1 Theoretical position

This study adopts social constructionism as its theoretical lens when understanding and analysing data.^35^ We believe that multiple realities and perspectives exist among researchers. These are subjective and socially constructed and thus depend on participants' cultural, political and historical backgrounds. Researchers (and thus their work) are shaped by their relationships with public contributors. From the social constructionism perspective, the dynamics of social interactions are essential to understand how new knowledge is achieved.^36^ Thus, in our analysis, we focused on the processes around PPIE rather than its structures.

Social constructionism can be used to justify a more collaborative form of inquiry.^35^ This can be achieved by conducting research together with the public contributors. Collaborative work can be seen among our participants who involve the public in their work but also in our project, as we involved two public contributors as co‐researchers.

### 2.2 Participants and data collection

Alongside big data researchers, we included facilitators of PPIE activities in big data projects. Facilitators (some of whom might be qualified researchers) are in charge of the overall organization of the PPIE progress; they co‐ordinate, organize and facilitate activities and act as intermediaries between researchers and public contributors.^37^ They often are recruited at research institutions to support specific big data research projects. Throughout the paper, for clarity, we will refer to both groups as researchers. All participants had to have an experience of involving or engaging seldom‐heard communities or aiming to reach them.

We recruited through Twitter, bulletins and established networks within big data research such as Health Data Research UK. Interested participants contacted the author for further details and to register their interests. Interviews were conducted on Zoom between March and June 2022. Interviews were later transcribed and anonymized, with all participants assigned pseudonyms.

A semistructured interview guide was developed to elicit participant experiences of PPIE with seldom‐heard communities. We also included an opportunity for them to speak about communities that they planned to reach or tried to engage but were unsuccessful. After the first interview, co‐authors met to reflect on the topic guide. One follow‐up question on what participants perceived as a seldom‐heard community was added to the topic guide.

Only limited demographics were collected from participants to protect their anonymity. Twelve participants took part in the study. We reached data saturation when no new themes appeared in our analysis.^38^ Participants were based in England (*n* = 5), Scotland (*n* = 4), Belgium (*n* = 2) and Canada (*n* = 1). The majority were women (*n* = 11) and there was one man. Their experience of research and PPIE ranged from two and a half years to 20 years, with an average of 9 years. We also asked them to describe themselves as researchers (*n* = 6) or facilitators (*n* = 9), although they could have chosen both options. Six participants were from an ethnic minority background.

### 2.3 Data analysis

We conducted a reflective inductive thematic analysis.^39^, ^40^, ^41^ This method allowed us to identify patterns across all interviews systematically. Thus, we unpacked the realities experienced by researchers. We used both semantic and latent coding. Semantic coding shows more explicit patterns within the data and stays as close as possible to what participants said. This allowed us to share specific practical examples of PPIE strategies. Latent coding provided more implicit and interpretive reflection on the data. Initially, one interview was coded jointly by three authors (P. T., S. A., N. T.) in Word. Then, the author (P. T.), an experienced qualitative researcher, inductively coded the remaining interviews, supported by NVivo 12. We met as a team on multiple occasions to discuss the data analysis and develop and refine further themes.

Public contributors can be meaningfully involved in qualitative analysis^42^ and trained to conduct reflexive thematic analysis.^43^ Two authors (S. A. and N. T.) who are public contributors received training in reflexive thematic analysis (focusing on being reflective, coding process and refining themes). They were involved in the designing the study, initial coding and developing and refining the themes. They are both experienced public contributors and S. A. also acts as the Data Ambassador for Care and Health Informatics theme within the Applied Research Collaboration North West Coast. This role involved raising awareness and knowledge about big data research. Two authors (S. A. and N. T.) also took part in an exercise reflecting on how their backgrounds influenced what they perceived in the data and what they brought to the analysis.

The research‐active authors also reflected on their academic backgrounds (P. T., K. F., S. E. R. and L. F.). Research team consisted of qualitative researchers with experience of involving and engaging the public, and those who conducted research in big data. These different perspectives allowed us to bring distinct views to the data analysis and furthered our understanding of the experiences of our participants.

## 3 FINDINGS

We present four themes that explore how researchers involved and engaged seldom‐heard communities in big data research: (1) abstraction and complexity of big data, (2) one size does not fit all, (3) working in partnership and (4) empowering the public contribution. All themes appeared throughout all interviews, which provides an indication that these experiences were commonly shared among participants (even if participants were based in different countries). We have provided additional quotes in Supporting Information: Appendix 1 that offer further examples of how the participants involved and engaged seldom‐heard communities around big data research.

### 3.1 Abstraction and complexity of big data

Big data can be an abstract and difficult topic to explain to the public. Participants said that conversations about big data include technical, specialist's vocabulary, jargon, references to legislation and regulations. Researchers found it challenging to discuss the complexity of this kind of research with public contributors in lay terms:

Big data is a really complex environment to navigate both in terms of the research, but also in terms of like the regulatory aspects and legislative aspects. (Sophia)

Sometimes, the difficulty in explaining big data research impacted on participants' experience of involving the public. Public contributors can have a role in advising (or deciding) if researchers may access routinely collected health data for research purposes. Here, the public contribute to the governance groups of these initiatives. Researchers who worked with these groups found it hard to explain to the public the purpose of big data research. They struggled to contextualize the concept of big data to the public if it did not directly refer to the public contributors' health condition or a topic that might interest them. The following extracts illustrate that challenge as the participant refers to bringing public contributors to support big data infrastructure:

Project (…) was just looking at the infrastructures of big data. It was really challenging to actually put that into a context that was relevant to members of the public; they kind of said ‘well we don't even know if you want us to be involved, we don't really see how we can be because this is all to do with linking up datasets with each other and it's all very technical, and it's not really anything to do with our living experience as patients or as members of the public’. So that was that was quite a hard project actually to think about. (Sienna)

It is not only public contributors who can be confused by big data jargon. Some participants who were not data researchers said that their familiarity with the topic was more akin to the public contributors rather than data researchers they worked with on the project. They might feel uncomfortable asking questions or requesting clarification. The public contributors often were more confident in asking these kinds of questions. This was seen as a very positive element of PPIE by a participant:

I'm sometimes really pleased when [public contributors] ask questions. Because I'm like oh, good, I don't know if I could have asked that, but so I'm really pleased that you did. I probably should have known that, but I don't, so I'm glad you asked it. (Robyn)

Participants felt that promoting the benefits of big data research, being transparent in how data are used and building trust with the public would ensure that some negative media stories around big data research could be counteracted. They believed that overall, the general public would be supportive of data sharing to improve healthcare. They recognized the need for effective communication between researchers and the public. In individual projects, they suggested training and supporting the public contributors around big data research but described it as a slow and time‐consuming process.

One of the things that we really do is kind of work with our staff to make sure that they are able to explain it in kind of like plain English. If we were to have a session about something like trusted research environments, which can be kind of like a technical. Then we would work with staff to actually plan the presentations (…) to make sure that the language is right, we also hold drop‐in sessions once a month so that members of the public that we work with can come in and say ‘I have a question’. (…) And so we bring in some of our more technical staff because I have no technical knowledge myself. (Harriet)

Participants spoke about how communication must continue outside the research projects and involve the broader community. The public contributors involved in big data research are essential to helping further engagement with their communities. As they become more familiar with big data research, their knowledge can be utilized to engage with the general public and raise awareness of big data research. They can help explain what big data research is about, its benefits and how it works. Here, a participant speaks about explaining in lay terms a technical term related to data:

When it comes to data and infrastructure and things, it can be very complex. There's lots of big words like pseudonymisation [laughing] and things like this, so we worked with the public members to create this animation, which gives a snapshot of what the project's like and it's an accessible snapshot. (Robyn)

This theme shows that talking about big data can be complex and challenging. However, there was an agreement that PPIE around big data research takes the researcher away from numbers and allows them to bring a human face to the data. The following excerpt explains this:

I love doing this type of analysis of, you know, hitting the buttons and seeing the graphs come up and seeing results. It's really exciting, but you miss that contact with people. And having that PPI group, there was a really good way for me to touch base and think about what the numbers meant. And think about the stories behind some of the data. And connect it to people's lived experience and I think that's really important. (…) To me, it's ones and zeros, but in reality that one is death. So it's really important to have that in front of your mind, and I think that brings it home when you've got a group of people in front of you who are really interested in what you're doing and to whom it could potentially make a difference. (Zoe)

### 3.2 One size does not fit all

This theme elicits the need for researchers to be flexible and often innovative when involving public contributors in big data research. Participants did not have one prescription on how to successfully work with the public contributors.

How PPIE looked in the participants' work differed based on the project needs, public interest or experiences. Public contributors can be involved in different roles within projects around big data research. These included contributing to the review of the data access process and as co‐investigators or members of advisory groups for specific projects. The following quote shows how public contributors can assist with decisions over whether and how researchers can access routinely collected medical data for research purposes.

That's a group of around eight members of the public who we meet with on a quarterly basis to get their views on our kind of engagement plans (…) and also to get them to become more part of our project approval process is something they've been really keen to do, so we're looking at our kind of review process. Researchers who want access to routinely collected health and social care data puts their applications in and it goes through a rigorous, multistage approval process and one of those that we're looking to do is to have the public voice within that so their vote, their part of it would be an assessment of the public value of the projects that come in. (Alex)

Participants said that public contributors can have a much more active role and co‐share responsibilities with researchers:

We have two co‐leads. One of them is myself and but the other one is a member of the public, so that from the very beginning, I am working very closely with [the public contributor] so that we can kind of shape this programme together, making sure that the public views are fed in right from the very beginning and as part of that we've also got a leadership (…) and so in this leadership team, it's half public contributor, patient‐public contributors and the other half would be kind of like professionals such as myself. (Harriet)

How to work with each community might depend on their needs. Many participants spoke about the need to understand the specific community that they were planning to work with. Here, a participant suggests a pre‐engagement engagement to understand what PPIE should look like:

It's just really interesting about doing that pre‐work to set up the scope and the scale of the engagement work and then to set up the environment that would be the safe as possible, so it's almost like a pre‐engagement engagement where you're really setting up the safe environment to allow for good public engagement to happen for diverse members. (Victoria)

Who represents seldom‐heard communities differed among participants. Participants often spoke about aiming to be reflective of the community. However, they recognized that it was not always possible (or feasible) to reach everyone who might potentially contribute. They admitted that because of their recruitment methods, limited resources or time, the public contributors who were generally involved often represented a limited range of demographics. Each community is different and might require different PPIE strategies. They argued that the recruitment should be specifically tailored to the group they wanted to reach. The communities that were most often involved in PPIE were generally white and elderly. The seldom‐heard communities they wanted to involve included ethnic minorities, people experiencing homelessness, traveller communities or different age groups (especially younger people). However, they also wanted to reach people with particular health conditions or improve male representation. The following quote illustrates how participants perceived their role in encouraging diversity:

We do try to reach out to seldom‐heard groups. We are currently undertaking an audit of our group to see how, where we're lacking, 'cause I suppose within the patient and public involvement there tends to be a certain type of person who volunteers and has got the time. So tend to be retired, tend to be white more often than not, and so we are keen to widen our demographic (…) we're not just interested in ethnicity (…) it tends to be quite a lot of women as well that volunteer, so you know, increasing, men, also increasing our younger population. (August)

### 3.3 Working in partnership

PPIE is not conducted in a silo. The participants worked with others (organizations, charities, public services and public contributors) with the aim of being inclusive and to reach more diverse communities, especially around big data research. This theme explores these different actors' roles in successful PPIE.

These partnerships have the potential to fill the gaps in researchers' understanding of local communities. Some participants recognized that researchers themselves could be a hard‐to‐reach group. Meetings can be held during working hours or be otherwise inaccessible to public contributors. Others recognized that the diversity of their teams is important and might reflect how well they involve and engage communities.

I think while we don't have as much diversity as we could in our staff, it's harder for us to communicate or share those messages or understand the groups that we're trying to reach. (Arabella)

Charities and organizations already provide existing links with the community and offer that bridge for researchers to reach the seldom‐heard groups. They can assist with recruitment and engagement strategies. However, there is a risk that a researcher will not necessarily improve the diversity of their group but rather take over the demographic composition of the group they engaged with, as this participant explains:

So it was mainly about because I was kind of piggybacking on a charity, on several charities groups. It was down to who they had picked up and they were already actually meeting via Zoom this charity, so I kind of inherited their diversity or degree of diversity. (Zoe)

However, as much as these partnerships can be helpful, establishing them is not easy. It can be time‐consuming to build that trust with the charity, and participants recognized that this needs to be an ongoing relationship that should benefit both parties.

Some participants also said that that relationship could be confusing to the potential public contributors if there is more than one research team working on that project (and thus trying to involve them). The following extract shows how one of the participants struggled to get some patient groups involved because they already had been working with other researchers:

I contacted several [patient groups] in [the city] to see if they would be interested in doing some PPI workshops with them or telling them a bit more about the research we're doing. (…) They didn't necessarily know that they it was the right thing for them at the time, but also they'd had so many researchers getting in contact with them that it's they said it's just really difficult for us to choose who we work with and if they've already got a relationship with somebody else. Then they may choose to work with them obviously instead. (Sienna)

Researchers can act as facilitators of PPIE or bring in trained experts (who might not necessarily be familiar with big data research). The facilitators' role is to act as this connecting bridge during work, an intermediary between researchers and the public contributors.

What we are trying to do is bring these people on board and explain to us what it is, and we try to turn it into more lay language and sometimes with [public contributors], engage them to have a conversation so that they can actually challenge the experts rather than us doing it. So we are more of an inbetweener in that sense. (Kimberly)

PPIE is also about involving individual public contributors. Participants often spoke about how interested and passionate public contributors can become about their involvement. These partnerships require working together and respecting each other. Some participants spoke highly of public contributors they worked with:

And one thing that I think that is often forgotten is about [public] members is that they are just, they're not just patients or they're not just a member of the public. These are very talented, very skilled people. You know they've got their own life skills. You know they've got their own careers. They've got all of the skills and knowledge from that, and I think it's great that they want to volunteer with us and help share some of that. (Robyn)

Only when truly working in partnership with public contributors can it lead to their empowerment. This is the focus of the next theme.

### 3.4 Empowering the public contribution

Participants felt that for involvement to be successful, there must be a power balance between researchers and the public contributors. Empowerment gives public contributors the ability to contribute to the involvement process fully. This can be achieved through ongoing support and ensuring that they become more familiar with big data research or projects that they are involved in. As the following qoute illustrates, this is a continuing process.

Giving a sort of chance for people to ask questions, which was the nice thing about that project is that it wasn't a one‐off, people could go away, look up something for themselves and then they could come back and be like what's this and they'd post a link and then we'd come back and answer those questions. So it was quite a nice kind of two‐way in that sense. (Drew)

Most participants felt that public contributors need to be supported at each stage of the involvement process but also recognized that this can be time‐consuming and requires additional work. Some suggested an open‐door policy where public contributors could reach researchers anytime and thus also feel like a part of the team. WhatsApp groups for public contributors can be a safe place to discuss the project further. Public contributors should receive training or induction both around the project and PPIE (especially if they are involved in a research project for the first time). One of the techniques that supported the public in understanding the jargon around big data research was a ‘live dictionary’, which could be updated as people asked questions throughout the lifetime of the research project.

But one of the things that we've created is an ongoing glossary. And if there's any words or phrases that the [public] members don't understand, it's a case of pop it into that glossary, and someone will answer it for them. (Robyn)

However, participants recognized that not all training can be equally helpful and that some institutional resources were more bureaucratic and could potentially discourage people from being involved. This is illustrated by the following quote talking about the focus on training offered by the academic institution to new public contributors involved in the research:

[The training] is quite formal and it's about like the whole university obviously it's not about big data, it's not really keyed towards seldom like heard groups or different types of groups, so I think there's other types of training that could still be useful for people, even if it's just, you know, stories of being involved that are from people who are more like them. So I think it could be a little bit of a little bit tailored, and some of it's very dry if I'm honest. (Zoe)

After receiving all this training and support, some participants felt that there is a danger that the public contributors start offering more of an expert view rather than a lay person perspective. There is a fine balance between understanding the project enough to be able to provide a nuanced contribution and where public contributors become what one can describe as ‘usual suspects’ of people who keep getting involved and thus become more like professionals. One participant spoke of a successful approach to dealing with this challenge:

it is a really fine line between building their knowledge to get involved and becoming an expert in that and kind of losing that public perspective (…) to kind of help with that; we do also have members of the public in a role for only specific amount of time. So, for example, now [advisory board]. They're only there three years, and then we kind of refresh the board, so with that, we're constantly bringing in that kind of like newer public perspective as well. (Harriet)

Empowerment must be felt in practice and involvement needs to be genuine. Public contributors must feel that they make a difference. In the ‘one size does not fit all’ theme, a researcher spoke about the public contributors' panel assessing if researchers can access medical data for research purposes. The participant described how the public contributors perceived this and how it could be expanded for more empowerment:

‘Do you agree with our decisions over whether these were approved or not?’ And in the main, they aligned with what the decisions had been, but on a couple of occasions, they were like ‘we don't see the public value in doing this. It's not well explained’, so is either it wasn't when explained or the public value wasn't there, and so that going more of a point of challenge for them and made it quite clear that they wanted to be part of the genuine process of review. (Alex)

Participants pointed out that only when there is a real sense of empowerment can public contributors' involvement impact positively on the research projects. There are multiple ways by which public contributors can shape projects. Participants named the following contributions: ensuring that the research questions address the public interest, co‐analysing study results, advising if researchers' ideas and thoughts are on the right track (e.g., appropriate wording used or right engagement strategy put in place) and public contributors doing sense‐checking and contributing to potential engagement strategies with the broader public. The following quote shows the variety of involvement and its impact:

Extremely impactful, (…), it's actually led to changes in the direction of our work, but in cases where that hasn't necessarily happened, that they've been more supportive of what we're kind of thinking and it has changed the way that some are kind of like thinking about the topic of public trust and public confidence, for example, and we only ever used to think like the wording that we would use as an organisation was we need to earn public trust. We need to build public trust but then through the [advisory board] through exploring that a bit more, we've kind of changed our way of thinking, so it's more about demonstrating trustworthiness in the use of data and building public confidence. (Harriet)

This theme has shown that public involvement should not be an afterthought and needs to be a genuine (but often time‐ and resource‐consuming) process that can have a significant impact on researchers' work. This can be especially seen in the following extract:

It is difficult to do really well, and it takes a lot of time and a lot of resources, and I think people underestimate that. I also think there's a culture towards PPI as a tick box. (Penelope)

## 4 DISCUSSION

Our findings have shown that talking about big data ‘with’ (rather than ‘to’) public contributors can be challenging, but that PPIE can be meaningful for both researchers and public contributors. The findings elicited how researchers and their research can benefit from involving and engaging seldom‐heard communities. Table 1 summarizes the key recommendations. This adds to the previous literature on meaningfully including a diverse range of communities^44^ and is relevant to other areas of health and social care research. PPIE requires time and resources,^45^ and not all communities are often equally involved.^46^ However, our participants have shown that inclusion around big data research (because of the complexity of the topic) takes additional time and resources to succeed (even in contrast to other health research). This can be seen in extra activities such as a ‘pre‐engagement engagement’, which was suggested as a baseline for successful working with the community. Our findings challenge the perspectives of some researchers who believe that public contributors rarely care about or can understand big data research and thus are not able to be involved in decisions around whether medical data can be reused for research.^47^ Involving and engaging seldom‐heard communities in big data might be more challenging than in other forms of health research but it is important as big data research offers an opportunity to reduce health disparities.^48^ Without seldom‐heard voice input, this might not happen.

**Table 1:** Key recommendations on involving and engaging seldom‐heard communities around big data research.

1. Provide information in lay language and, where not possible, explain in simple English. Ensure that these explanations are available at any point to the public contributor (e.g., through an online dictionary).
2. Rotate public contributors on a ‘big data panel’ every 3 years to bring in new ideas and lay perspectives.
3. Reach out to new communities for at least 50% of the new attendees, potentially using charitable/partner organizations to help.
4. Identify relevant seldom‐heard communities for each project.
5. Consider strategies to add additional diversity on multiple characteristics (e.g., LGBTQ+ and ethnic minority, or disability).
6. Adequate and ongoing training/support for PAs should be provided to empower them so that they can truly contribute.

The findings confirm that defining a group as a seldom‐heard group is context‐specific.^33^, ^49^ The participants named numerous types of seldom‐heard communities involved and engaged within the context of their work. Researchers should reflect on who would be the most seldom‐heard group within the context of their study and recognize that this might include more than one community. The concept of superdiversity^50^, ^51^ could provide researchers with further guidance on moving away from looking at a single characteristic (e.g., ethnicity) of the community and focusing instead on diversity within diversity. This would ensure that the needs of communities within communities are considered.

Researchers need to take time to plan PPIE well as they design their projects. NIHR guidance^33^, ^52^ recognizes this and recommends working with communities on a long‐term basis. Our findings have shown the importance of building and maintaining relationships with organizations, especially charities. This confirms previous research that shows that links to the third sector are crucial in building trust.^53^, ^54^, ^55^ They often act as gatekeepers but also have the potential to act as a partner. There is, however, a risk that researchers would not reach many communities as they might be limited to the partner organization's level of diversity.

There is a growing trend to establish a pool of volunteers interested in participating in PPI activities.^56^ This approach might appeal to those who have time, resources and feel comfortable with working with institutions. However, this risks public contributors becoming ‘usual suspects’ of people who are involved regularly and thus not providing new contributions. There is the danger that they will become more expert than researchers themselves, thus no longer providing lay experiences and views in the project. There remains a contentious issue: how to strike a balance between public contributors being capable of contributing fully but also retaining a lay perspective.^57^ One of our participants suggested the need to change public contributors on advisory boards every 3 years. This offers a solution to deal with the challenge of ‘usual suspects’ and brings a fresh public perspective but adds more work on the part of the researchers to recruit, provide training and support new public contributors on the project. The other option is to sense‐check any work with the broader public.

Researchers should also ensure that any involvement is not tokenistic and enables power‐sharing between researchers and the public contributors.^21^ There is no one ‘right’ way to do it, and the approach depends on the project's needs (or resources) and the public contributors' interests. However, their interests should not be confused with their understanding of the topic, and researchers should provide training to improve public contributors' knowledge, thus facilitating their ability to contribute. This genuine empowerment was seen as crucial among our participants when discussing big data research with public contributors. Although not mentioned by our participants, some public contributors, for example, coming from Indigenous communities, might also require researchers to respect their values to feel truly empowered.^58^

## 5 STUDY LIMITATIONS

The study participants came from diverse communities, for example, various ethnic minority backgrounds. However, we did not record if they are a part of other seldom‐heard communities, for example, LGBTQ+ or people living with disabilities. We only explored the perspectives of the researchers, and there is a possibility that the public contributors (including those coming from seldom‐heard communities) would have a different view on their PPIE activities around big data research. As big data is a fast developing and diverse research area, new ways of involving and engaging will emerge, so future research should further explore how researchers involve and engage public contributors and how concepts of super diversity could be utilized.

## 6 CONCLUSION

Our study explored how researchers involve and engage public contributors (especially seldom‐heard communities) in a meaningful way in big data research. The findings highlight that there is no one right approach to doing PPIE and that PPIE strategies are project‐specific and depend on the public contributors, researchers' needs, resources and time available. We encourage others to reflect on their involvement strategies and hope that these results will support researchers who want to involve more seldom‐heard communities in complex research topics such as big data.

## CONFLICT OF INTEREST STATEMENT

The authors declare no conflict of interest.

## ETHICS STATEMENT

We received ethical approval to conduct this study from the ethics committee at University of Liverpool under the number 10063.

## Supporting information

Supporting information.

## Data Availability

Anonymized data are available upon reasonable request.

## References

[1] NHS .UK Policy Framework for Health and Social Care Research. NHS; 2020.

[2] Mockford C , Staniszewska S , Griffiths F , Herron‐Marx S . The impact of patient and public involvement on UK NHS health care: a systematic review. Int J Qual Health Care. 2012;24(1):28‐38. 22109631 10.1093/intqhc/mzr066

[3] Sofolahan‐Oladeinde Y , Newhouse RP , Lavallee DC , Huang JC , Mullins CD . Early assessment of the 10‐step patient engagement framework for patient‐centred outcomes research studies: the first three steps. Fam Pract. 2017;34(3):272‐277. 28334775 10.1093/fampra/cmx013

[4] Lalani M , Baines R , Bryce M , et al. Patient and public involvement in medical performance processes: a systematic review. Health Expect. 2019;22(2):149‐161. 30548359 10.1111/hex.12852 PMC6433319

[5] Aitken M , Tully MP , Porteous C , et al. Consensus statement on public involvement and engagement with data‐intensive health research. Int J Popul Data Sci. 2019;4. 10.23889/ijpds.v4i1.586 PMC8142968 34095528

[6] Glasby J , Beresford P . Commentary and issues: who knows best? Evidence‐based practice and the service user contribution. Crit Soc Policy. 2006;26(1):268‐284.

[7] Tritter JQ . Revolution or evolution: the challenges of conceptualizing patient and public involvement in a consumerist world. Health Expect. 2009;12(3):275‐287. 19754691 10.1111/j.1369-7625.2009.00564.x PMC5060496

[8] NIHR . Briefing notes for researchers—public involvement in NHS, health and social care research. 2021. Accessed July 14, 2022. https://www.nihr.ac.uk/documents/briefing-notes-for-researchers-public-involvement-in-nhs-health-and-social-care-research/27371

[9] Mehta N , Pandit A . Concurrence of big data analytics and healthcare: a systematic review. Int J Med Inform. 2018;114:57‐65. 29673604 10.1016/j.ijmedinf.2018.03.013

[10] Taylor M . Information governance as a force for good? Lessons to be learnt from Care.data. SCRIPTed. 2014;11.

[11] Aitken M , de St. Jorre J , Pagliari C , Jepson R , Cunningham‐Burley S . Public responses to the sharing and linkage of health data for research purposes: a systematic review and thematic synthesis of qualitative studies. BMC Med Ethics. 2016;17(1):73. 27832780 10.1186/s12910-016-0153-x PMC5103425

[12] Hays R , Daker‐White G . The care.data consensus? A qualitative analysis of opinions expressed on Twitter. BMC Public Health. 2015;15(1):838. 26329489 10.1186/s12889-015-2180-9 PMC4556193

[13] Muller SHA , Kalkman S , Van Thiel GJMW , Mostert M , Van Delden JJM . The social licence for data‐intensive health research: towards co‐creation, public value and trust. BMC Med Ethics. 2021;22(1):110. 34376204 10.1186/s12910-021-00677-5 PMC8353823

[14] Hill EM , Turner EL , Martin RM , Donovan JL . “Let's get the best quality research we can”: public awareness and acceptance of consent to use existing data in health research: a systematic review and qualitative study. BMC Med Res Methodol. 2013;13(1):72. 23734773 10.1186/1471-2288-13-72 PMC3682867

[15] Ford E , Boyd A , Bowles JKF , et al. Our data, our society, our health: a vision for inclusive and transparent health data science in the United Kingdom and beyond. Learn Health Syst. 2019;3(3):e10191. 31317072 10.1002/lrh2.10191 PMC6628981

[16] Dixon‐Woods M , Ashcroft RE . Regulation and the social licence for medical research. Med Health Care Philos. 2008;11(4):381‐391. 18633729 10.1007/s11019-008-9152-0

[17] Harrison JD , Auerbach AD , Anderson W , et al. Patient stakeholder engagement in research: a narrative review to describe foundational principles and best practice activities. Health Expect. 2019;22(3):307‐316. 30761699 10.1111/hex.12873 PMC6543160

[18] INVOLVE. Diversity and Inclusion: What's it About and Why is it Important for Public Involvement in Research? INVOLVE; 2012.

[19] Dawson S , Campbell SM , Giles SJ , Morris RL , Cheraghi‐Sohi S . Black and minority ethnic group involvement in health and social care research: a systematic review. Health Expect. 2018;21(1):3‐22. 28812330 10.1111/hex.12597 PMC5750731

[20] Flynn R , Walton S , Scott SD . Engaging children and families in pediatric health research: a scoping review. Res Involv Engagem. 2019;5(1):32. 31700676 10.1186/s40900-019-0168-9 PMC6827239

[21] Ocloo J , Matthews R . From tokenism to empowerment: progressing patient and public involvement in healthcare improvement. BMJ Qual Saf. 2016;25(8):626‐632. 10.1136/bmjqs-2015-004839 PMC4975844 26993640

[22] IPFCC. Diverse Voices Matter: Improving Diversity in Patient and Family Advisory Councils. IPFCC; 2018.

[23] Bonevski B , Randell M , Paul C , et al. Reaching the hard‐to‐reach: a systematic review of strategies for improving health and medical research with socially disadvantaged groups. BMC Med Res Methodol. 2014;14(1):42. 24669751 10.1186/1471-2288-14-42 PMC3974746

[24] Madden M , Speed E . Beware zombies and unicorns: toward critical patient and public involvement in health research in a neoliberal context. Front Sociol. 2017;2:7.

[25] Kumar BN , Hargreaves S , Agyemang C , James RA , Blanchet K , Gruer L . Reducing the impact of the coronavirus on disadvantaged migrants and ethnic minorities. Eur J Pub Health. 2021;31(suppl_4):iv9‐iv13. 34751368 10.1093/eurpub/ckab151 PMC8576303

[26] Nguyen TH , Cheah PY , Chambers M . Identifying ‘hard‐to‐reach’ groups and strategies to engage them in biomedical research: perspectives from engagement practitioners in Southeast Asia. Wellcome Open Res. 2019;4:102. 31723673 10.12688/wellcomeopenres.15326.1 PMC6823897

[27] Rayment J , Lanlehin R , Mccourt C , Husain SM . Involving seldom‐heard groups in a PPI process to inform the design of a proposed trial on the use of probiotics to prevent preterm birth: a case study. Res Involv Engagem. 2017;3(1):11. 29062536 10.1186/s40900-017-0061-3 PMC5611657

[28] Prinjha S , Miah N , Ali E , Farmer A . Including ‘seldom heard’ views in research: opportunities, challenges and recommendations from focus groups with British South Asian people with type 2 diabetes. BMC Med Res Methodol. 2020;20(1):157. 32539718 10.1186/s12874-020-01045-4 PMC7296709

[29] Tierney S , Dawson S , Boylan A‐M , et al. Broadening diversity through creative involvement to identify research priorities. Res Involv Engagem. 2021;7(1):3. 33407929 10.1186/s40900-020-00244-z PMC7787225

[30] Hanafin J , Lynch A . Peripheral voices: parental involvement, social class, and educational disadvantage. Br J Sociol Educ. 2002;23(1):35‐49.

[31] Silva DS , Smith MJ , Upshur REG . Disadvantaging the disadvantaged: when public health policies and practices negatively affect marginalized populations. Can J Public Health. 2013;104(5):e410‐e412. 24183183 10.17269/cjph.104.3895 PMC6974166

[32] Snow ME , Tweedie K , Pederson A . Heard and valued: the development of a model to meaningfully engage marginalized populations in health services planning. BMC Health Serv Res. 2018;18(1):181. 29544486 10.1186/s12913-018-2969-1 PMC5856315

[33] NIHR . Improving inclusion of under‐served groups in clinical research: guidance from INCLUDE project. 2020. Accessed November 5, 2020. https://www.nihr.ac.uk/documents/improving-inclusion-of-under-served-groups-in-clinical-research-guidance-from-include-project/25435

[34] Teodorowski P , Jones E , Tahir N , Ahmed S , Frith L . Public involvement and engagement in big data research: protocol for a scoping review and a systematic review of delivery and effectiveness of strategies for involvement and engagement. BMJ Open. 2021;11(8):e050167. 10.1136/bmjopen-2021-050167 PMC8378392 34413107

[35] Gergen KJ . An Invitation to Social Construction. 3rd ed. Sage; 2015.

[36] Burr V . Social Constructivism. Vol 3. Routledge; 2015.

[37] Todd S , Coupland C , Randall R . Patient and public involvement facilitators: could they be the key to the NHS quality improvement agenda? Health Expect. 2020;23(2):461‐472. 32022356 10.1111/hex.13023 PMC7104637

[38] Guest G , Bunce A , Johnson L . How many interviews are enough? An experiment with data saturation and variability. Field Methods. 2006;18(1):59‐82.

[39] Braun V , Clarke V . Using thematic analysis in psychology. Qual Res Psychol. 2006;3(2):77‐101.

[40] Braun V , Clarke V . Thematic Analysis: A Practical Guide. Sage; 2021.

[41] Braun V , Clarke V . Reflecting on reflexive thematic analysis. Qual Res Sport Exerc Health. 2019;11(4):589‐597.

[42] Garfield S , Jheeta S , Husson F , et al. Lay involvement in the analysis of qualitative data in health services research: a descriptive study. Res Involv Engagem. 2016;2(1):29. 29507764 10.1186/s40900-016-0041-z PMC5831865

[43] Hemming L , Pratt D , Bhatti P , Shaw J , Haddock G . Involving an individual with lived‐experience in a co‐analysis of qualitative data. Health Expect. 2021;24:766‐775. 33788373 10.1111/hex.13188 PMC8235892

[44] Taylor C , Gill L , Gibson A , Byng R , Quinn C . Engaging “seldom heard” groups in research and intervention development: offender mental health. Health Expect. 2018;21(6):1104‐1110. 30030880 10.1111/hex.12807 PMC6250876

[45] Boden C , Edmonds AM , Porter T , et al. Patient partners' perspectives of meaningful engagement in synthesis reviews: a patient‐oriented rapid review. Health Expect. 2021;24:1056‐1071. 34048618 10.1111/hex.13279 PMC8369105

[46] Beresford P . *Beyond the Usual Suspects: Towards Inclusive User Involvement: Findings*. Shaping Our Lives Publ.; 2013.

[47] Mouton Dorey C , Baumann H , Biller‐Andorno N . Patient data and patient rights: Swiss healthcare stakeholders' ethical awareness regarding large patient data sets—a qualitative study. BMC Med Ethics. 2018;19(1):20. 29514635 10.1186/s12910-018-0261-x PMC5842517

[48] Zhang X , Pérez‐Stable EJ , Bourne PE , et al. Big data science: opportunities and challenges to address minority health and health disparities in the 21st century. Ethn Dis. 2017;27(2):95‐106. 28439179 10.18865/ed.27.2.95 PMC5398183

[49] Paprica PA , Sutherland E , Smith A , et al. Essential requirements for establishing and operating data trusts: practical guidance co‐developed by representatives from fifteen Canadian organizations and initiatives. Int J Popul Data Sci. 2020;5(1):1353. 33644412 10.23889/ijpds.v5i1.1353 PMC7894384

[50] Bradby H , Green G , Davison C , Krause K . Is superdiversity a useful concept in European Medical Sociology? Front Sociol. 2017;1:17.

[51] Kirwan G . Superdiversity re‐imagined: applying superdiversity theory to research beyond migration studies. Curr Sociol. 2021;70(2):193‐209.

[52] NIHR . Next steps for partnership working with patients and the public: engagement report. 2021. Accessed July 28, 2022. https://www.nihr.ac.uk/documents/next-steps-for-partnership-working-with-patients-and-the-public-engagement-report/28566

[53] Steel R . Actively involving marginalized and vulnerable people in research. In: Lowes L , Hulatt I , eds. Involving Service Users in Health and Social Care Research. Routledge; 2005;27‐38.

[54] Morrow E , Boaz A , Sally BF . Seldom‐heard groups. In: Boaz A , Morrow E , Brearley S , Ross FM , eds. Handbook of Service User Involvement in Nursing and Healthcare Research. Wiley‐ Blackwell; 2011:106‐119.

[55] Nelson E , Burns F . Impact through engagement: co‐production of administrative data research and the approach of the Administrative Data Research Centre Northern Ireland. Int J Popul Data Sci. 2020;5(3):1369. 34007887 10.23889/ijpds.v5i3.1369 PMC8104064

[56] Grotz J , Ledgard M , Poland F . Patient and Public Involvement in Health and Social Care Research. Springer International Publishing; 2020.

[57] Ives J , Damery S , Redwod S . PPI, paradoxes and Plato: who's sailing the ship? J Med Ethics. 2013;39(3):181‐185. 22267385 10.1136/medethics-2011-100150

[58] Rowe R , Carroll SR , Healy C , Rodriguez‐Lonebear D , Walker JD . SEEDS of Indigenous population health data linkage. Int J Popul Data Sci. 2021;6(1):1417. 34212119 10.23889/ijpds.v6i1.1417 PMC8218891

