# Supplementary material for: ‘To me, it's ones and zeros, but in reality that one is death’: A qualitative study exploring researchers' experience of involving and engaging seldom‐heard communities in big data research
Source: Health Expect. 2023 Jan 24;26(2):882–91. doi: 10.1111/hex.13713 (PMC10010102; doi:10.1111/hex.13713)
Supplement: Supplementary file 1 — Supporting information. [file HEX-26--s001.docx]

**Supporting quotes of how seldom-heard communities were engaged in big data research according to theme**

**Theme 1**

*“There's lots of things that people might not have heard of before, even considered or understood that goes on behind the scenes and some very complex areas to discuss, and we've had a few things that have been quite difficult (…) like trusted research environments (…) So it's definitely taken time to build up the knowledge base of [public contributors], but we've had, for example, external speakers from other organisations that work with this quite often (…) we share training or webinars with [public contributors] that might be of interest and if we're going to have a discussion on a particular area, we'll tend to kind of give them some background reading or a paper from us or a video to go and watch or something to help build their knowledge and we also tried to have discussions over a couple of meetings that we don't just have one discussion about it and that's it. We try to kind of embed it in the process a bit earlier so that we can have a few discussions, so again gives them more of a chance to kind of get grips with it and understand a bit better.”* (Arabella)

*“This was at the request of the patient and public contributors on that particular project, they said they really wanted to get to grips with some of the statistical methods that we were using (…) they really felt that if they understood the methods, they might be able to have a more fruitful conversation with us about how they feel about the way that we're running the study and how we're designing the analysis and things like that. So we ran a session where the one of the analysts on the project and I worked with them on the presentation. They gave a presentation to the patient and public contributors which walked through everything from what the GP will put into their computer and when they're having a consultation with them right through to where that data goes, what it looks like. Obviously, it was all just anonymized examples. It wasn't actual, they couldn't see the actual data. Then how the different methods that they use to process it and how that method is applied in the in the specific project and the patient and public contributors said, ‘actually, the fact that you've taken the time to sit down and explain that to us and say specifically how it's going to be used in this project and was really useful’ and we had a actually a really interesting discussion about could you include this or do you exclude this group of patients (…) and actually they started to come up with things which were coming from their own experience because they understood enough from what they said they could really add to the conversation, so I think taking the time and making the effort, if the contributors asked for it was really helpful.”* (Sienna)

**Theme 2**

"*We went as wide as we could because we really did want geographical location as well as diversity (…) our key things were lived experience of health inequalities. Then we looked as to access to maybe wider networks and then making sure that we had ethnic diversity across the range. We were able to recruit people from African backgrounds, Asian backgrounds and then we have some members who are white but have a different religion so Jewish for example and also [health conditions] (…) we've got quite a range, a diverse range of people, but we do recognise that the one individual cannot represent the whole population*"(Penelope)

*“If you want to hear from these groups, then you need to reward them and you should reward anyway, but you know, obviously resources are slim (…) it's about picking your targets, making sure that what you're doing suits the study that you're looking for. Because you know we don't have infinite resources, so you have to basically use them wisely. And if it's a study that is important to seldom heard groups, then you know you would need to target that and put your resources into that, and that's like finding out where they are, what they do, what things are important to them and going out to them there.”* (August)

**Theme 3**

*“There are links to other organisations, for example, [local charity working with seldom-heard community], and so we've had a really positive conversation with them and there could be mutually beneficial opportunities for us to open the invitation to people that they support to be part of our [public involvement group] and in turn it kind of supports their personal development programmes that they do for people (…) so we get the benefit of expanding the perspectives that are included within our group, but hopefully that's a useful thing for their personal development as well as part of the programmes that are doing underneath the charity umbrella.”* (Alex)

**Theme 4**

*“I think to facilitate good discussion. I think that you want no more than like 10 people. Because otherwise you can't include everybody and people become observers to a more dominant group, so (…) I think what works well for and even face-to-face, we would do like a smaller group so to ensure that everybody can contribute.” (August)*

*“They become experts, but you can't help that if you're in that realm. We have ways of mitigating that, and might be that naturally members leave, then you recruit newer members. But then there's so many challenges around that it's training. There has to be a level of training and understanding and does that make just because somebody got some knowledge. Does that make them make their opinion less worthy? I think, as long as we sense check it to the wider population and go out to community groups.* (Penelope)
